# Does Acellular Dermal Matrix Thickness Affect Complication Rate in Tissue Expander Based Breast Reconstruction?

**DOI:** 10.1155/2016/2867097

**Published:** 2016-04-12

**Authors:** Jessica F. Rose, Sarosh N. Zafar, Warren A. Ellsworth IV

**Affiliations:** Division of Plastic Surgery, Department of Surgery, Houston Methodist Hospital, Medical Office Building, 118400 Katy Freeway, Suite 500, Houston, TX 77094, USA

## Abstract

*Background*. While the benefits of using acellular dermal matrices (ADMs) in breast reconstruction are well described, their use has been associated with additional complications. The purpose of this study was to determine if ADM thickness affects complications in breast reconstruction.* Methods.* A retrospective chart review was performed including all tissue expander based breast reconstructions with AlloDerm (LifeCell, Branchburg, NJ) over 4 years. We evaluated preoperative characteristics and assessed postoperative complications including seroma, hematoma, infection, skin necrosis, and need for reintervention. We reviewed ADM thickness and time to Jackson-Pratt (JP) drain removal.* Results*. Fifty-five patients underwent 77 ADM-associated tissue expander based breast reconstructions, with average age of 48.1 years and average BMI of 25.9. Average ADM thickness was 1.21 mm. We found higher complication rates in the thick ADM group. Significant associations were found between smokers and skin necrosis (*p* < 0.0001) and seroma and prolonged JP drainage (*p* = 0.0004); radiated reconstructed breasts were more likely to suffer infections (*p* = 0.0085), and elevated BMI is a significant predictor for increased infection rate (*p* = 0.0037).* Conclusion*. We found a trend toward increased complication rates with thicker ADMs. In the future, larger prospective studies evaluating thickness may provide more information.

## 1. Introduction

Implant based breast reconstruction is the most common type of breast reconstructions performed in the United States [1]. According to the American Society of Plastic Surgery, 83,149 implant based breast reconstructions were performed in 2014 (81.3% of breast reconstructions), with 74,694 utilizing tissue expanders (73.1% of all reconstructions) [2]. Implant based reconstruction may be chosen because of faster recovery, lack of donor site morbidity, or patient comorbidities that may preclude autologous reconstruction [3]. Implant based breast reconstruction often requires placement of a tissue expander (TE) to enlarge the mastectomy skin envelope enough to fit the desired size of breast implant and ensure successful survival of the often thin mastectomy flaps. In the senior authors' practice, tissue expanders are often used after mastectomy as a bridge to autologous reconstruction, especially when there is a possibility of needing adjuvant therapy including postmastectomy radiation therapy (PMRT). Tissue expanders are most commonly placed in the subpectoral plane with coverage of the lower and lateral poles of the expander with acellular dermal matrices (ADMs) [1].

ADMs are biologic material initially used in revision breast surgery to fix contour deformities, rippling, and malposition. Their use in tissue expander based breast reconstruction has grown exponentially over the past decade. The ADM is placed in the inframammary and lateral mammary folds as a sling to reinforce and support the expander or implant [3–8]. It aids in covering the lower pole of the TE while supporting the position of the prosthesis, shaping the breast, and preventing device exposure in the setting of mastectomy flap necrosis [1, 6, 7]. ADM coverage of the lower pole helps to recreate lost anatomic landmarks after mastectomy, provide support, and allow for increased intraoperative fill volume [7–9]. It may also help prevent the formation of a capsule by decreasing local inflammation [10]. Despite these benefits, their use may increase complication rates, particularly regarding seroma [5]. There are many types of ADM products on the market; however AlloDerm (LifeCell, Branchburg, NJ) is the most commonly used product in the senior authors practice and the United States today [4].

In contrast, total submuscular placement results in the expander preferentially filling the superior pole, creating a less natural appearing breast. To create a natural, ptotic breast shape, the inferior pole of the expander can be left without muscle coverage, leaving a significant exposure risk [3]. Incorporating ADM can help recreate natural breast structure and ptosis, while simultaneously providing suitable expander coverage. Typically, the use of an ADM also allows the expander to be filled to a larger volume intraoperatively, decreasing requirements for postoperative visits and expansions [1, 3, 10]. Greater initial fill translates to fewer expansions and less time until definitive reconstruction with an implant or flap [5, 11]. Some authors feel that the use of ADM instead of total submuscular placement decreases postoperative pain and pain during expansion, although a randomized trial by Nguyen et al. failed to substantiate that claim [5, 11]. Other advantages include a suggested decreased capsular contracture rate, less revisions, and overall improved aesthetic outcome [5]. Hanna et al. compared expander based reconstruction with ADMs versus total submuscular placement including a patient satisfaction survey [12]. They showed higher mean scores for the ADM group regarding overall satisfaction, shape of the reconstruction, and ease of the expansion experience [12].

Anecdotally, we felt that patients in whom thicker ADMs were used were more likely to develop seromas or prolonged Jackson-Pratt (JP) drain output. Thus, we decided to evaluate our data retrospectively to delineate causation. To our knowledge, there have been no studies specifically looking at ADM thickness and development of complications (particularly seroma) or prolonged drain times.

## 2. Methods

We retrospectively analyzed records of all consecutive patients over approximately a four-year time period, from January 1, 2011, through April 1, 2015, who underwent breast reconstruction utilizing tissue expander and ADM by the senior author at our institution. Ninety percent of the mastectomies were performed by one of two fellowship trained breast surgical oncologists, with whom the senior author routinely collaborates. We included only those patients who were reconstructed with AlloDerm (LifeCell, Branchburg, NJ) and only included data of one plastic surgeon for consistency in technique of ADM, drain placement, and postoperative drain management and removal.

Multiple factors in the study group were examined: patient age, body mass index (BMI), presence of diabetes, smoking status, postoperative radiation treatment, development of complications (seroma, hematoma, infection, and skin necrosis), need for reintervention, time to Jackson-Pratt (JP) drain removal, thickness of the ADM, and eventual outcome (final breast reconstruction with DIEP flap or implant). Seroma and hematoma were both clinically defined as increasing breast size with fluid collections containing either serous fluid or blood, respectively. Infection was defined by the need for antibiotics, whether oral or intravenous, as determined clinically by the senior author. JP drain removal time was rounded to the nearest week and averaged per drain. We defined “thick” ADM as greater than or equal to 1.2 mm and “thin” ADM as less than 1.2 mm in thickness. This thickness was chosen, as it was the mean and median thickness of products utilized.

The aim of the study was, first, to assess differences in seroma rate and JP drain time in patients with thick versus thin ADMs. We postulated that thicker ADMs would produce more fluid, prolong integration time, and therefore lengthen time until drain removal. Second, we assessed complication rates between the two groups and risk factors for complication development (radiation, BMI, diabetes, and smoking).

### 2.1. Operative Technique

Our operative technique is as follows. The patient undergoes a mastectomy by the breast surgeon. The pectoralis major is elevated off of the chest wall using Bovie electrocautery and is disinserted to the 3 o'clock or 9 o'clock position, depending on laterality. AlloDerm is soaked for 10 minutes in a bacitracin and normal saline bath, gloves are changed, and the surgeon then places the ADM in the mastectomy cavity. It is sutured in place using interrupted 2-0 polydioxanone (PDS; Ethicon US, LLC, Somerville, NJ) from the 3 o'clock to 9 o'clock position. Tissue expanders are prepared on the back table and soaked in triple antibiotic solution. The operating team's gloves are exchanged for new gloves. The air from the TEs is removed and the expander is filled with 150 cc of injectable saline with methylene blue. The expander is placed under the pectoralis muscle and ADM, and its tabs are sutured in place using 2-0 PDS “U” stitches. The ADM and muscle are sutured together using 2-0 PDS for total expander coverage. Two 15-French round JP drains are placed: one deep to and one superficial to the ADM. If possible, we add more fluid to the expander until the cavity is filled with minimal tension on the mastectomy closure. We use the SPY Elite (NOVADAQ Technologies Inc., Huntington, NY) with indocyanine green to assess the viability of the mastectomy flaps when concern arises over perfusion. Once complete, we debride the edges of the wound to healthy tissue and close the skin using interrupted 3-0 poliglecaprone 25 (Monocryl; Ethicon US, LLC, Somerville, NJ) deep dermal and 4-0 poliglecaprone 25 subcuticular sutures, followed by Dermabond. Patients are admitted for 23-hour observation for pain control and drain care teaching. They are kept on appropriate antibiotic prophylaxis for 7 days.

All patients had at least 2 drains placed per breast (those with an axillary dissection had a 3rd drain in the axilla). Patients were seen in the office at least weekly until the drains were removed. Drains were removed when the output remained less than 30 cc per day, but their drains were removed by week 5 regardless of the output to decrease retrograde infection potential. All drains were dressed with Biopatch covered with Tegaderm, and this dressing was changed weekly.

### 2.2. Statistical Methods

Data for continuous variables are reported as the mean ± standard deviation. The Kolmogorov-Smirnov test was used for testing normal distribution of continuous variables. The independent Mann-Whitney test was used to compare the average drain times between groups of patients with different characteristics or complications. Between-group differences for dichotomous variables, including number of patients with DM, number of patients with radiation, number of smokers, number of patients with thick ADMs (thickness was dichotomized to thick and thin using cutoff of 1.2 mm), number of patients who developed seroma, hematoma, infection, and skin necrosis, number of patients who needed reoperation, and number of patients with complications, were examined using analysis of variance and the Fisher exact test or *χ* ^2^ test, as appropriate.

To identify which factors may affect average drain time, we used a linear regression model with average drain time as a dependent variable and age, BMI, DM, radiation, and smoking as independent variables. Also, to identify independent predictors for presence of complications at the conclusion of the study, we used a logistic backward regression model with presence of complications as the dependent variable and the independent variables mentioned above. A *p* value of ≤0.05 was considered statistically significant. Statistical analyses were performed using R version 3.1.3 software (Bell Laboratories, Madison, WI).

## 3. Results

Over a 4-year time period, 55 patients underwent 77 ADM/tissue expander breast reconstructions using AlloDerm. Patients' ages ranged from 23 to 76 (average 48.1) with an average BMI of 25.9. Five patients (6.5%) of the population were diabetic, 10 (13.0%) patients were smokers, and 20 (26.0%) patients required radiation treatment.

ADM thickness ranged from 0.86 mm to 2.18 mm (average 1.21 mm; median 1.21 mm). We defined thick ADM as 1.2 mm and above. Forty-one breasts were reconstructed with thick ADMs, while 36 breasts were reconstructed with thin ADMs. Further analysis to determine if the threshold for thick ADMs did not yield statistical significance for any value, so we maintained our original definition of a thick ADM.  Table 1 shows patient characteristics with thin versus thick ADMs, showing well-matched groups with the exception of diabetes.

Complications were more prevalent in the thick group, although not statistically significant (Table 2). We looked at our patient population for the development of complications by ADM thickness and by risk factor, mainly to make sure that our patient population behaved as predicted.

We compared patient characteristics to see how they impacted the development of complications. Smokers were more likely to develop skin necrosis (*p* < 0.0001) and require reintervention (*p* = 0.0064) than other patients. Diabetic patients were more likely to be older, had higher BMIs, and did not have thick ADMs. Patients who were radiated after expander placement were more likely to develop infections (*p* = 0.0085).

We also compared groups by presence or absence of complications. Presence of seroma was a risk factor for prolonged JP drainage (3.53 weeks versus 2.28 weeks, *p* = 0.0004) and was also a risk of other complications such as hematoma (*p* = 0.0092), infection (*p* = 0.0002), skin necrosis (*p* = 0.0064), and the need for reintervention (*p* < 0.0001) (see Table 3).

Patients with and without infections were also compared. Significant risk factors for infection included increased BMI (25.3 versus 30.2 with infection, *p* = 0.0037), radiation (60% of patients who were infected were radiated, *p* = 0.0085), hematoma (the one patient with a hematoma developed an infection, *p* = 0.0091), and seroma (50% of infected patients had seromas versus 7.5% who did not, *p* = 0.0002). Those with infection were also more likely to have prolonged drain times (2.28 weeks versus 3.50 weeks, *p* = 0.0001) and require reintervention (60% of infected patients versus 5.8% of those without infections, *p* < 0.0001). Younger patients were more likely to have developed an infection, average age 41.3 with infections versus 49.1 (*p* = 0.0273).

Patients who did and did not develop skin necrosis were also compared. Smoking was a significant risk factor (*p* < 0.0001). Patients with skin necrosis were prone to other complications like seroma (*p* = 0.0064) and hematoma (*p* = 0.0092) and were more likely to require reintervention (*p* < 0.0001). As anticipated, those requiring reoperation were more likely to be smokers (*p* = 0.0064), have seromas (*p* < 0.0001), hematomas (*p* = 0.0092), and infections (*p* < 0.0001), and require drains longer (2.33 weeks versus 3.20 weeks, *p* = 0.0024).

When ADM thickness was evaluated as a continuous variable, there was no significant threshold for the development of complications. However, patients with thicker ADMs were more likely to have infections (*p* = 0.0178) and skin necrosis (*p* = 0.0046) and require reoperation (*p* = 0.0022). Those with an elevated BMI were more likely to have infections (*p* = 0.0035) and skin necrosis (*p* = 0.0279), and BMI was the only significant risk factor for prolonged drain times (*p* = 0.0136).

**Figure 1:**
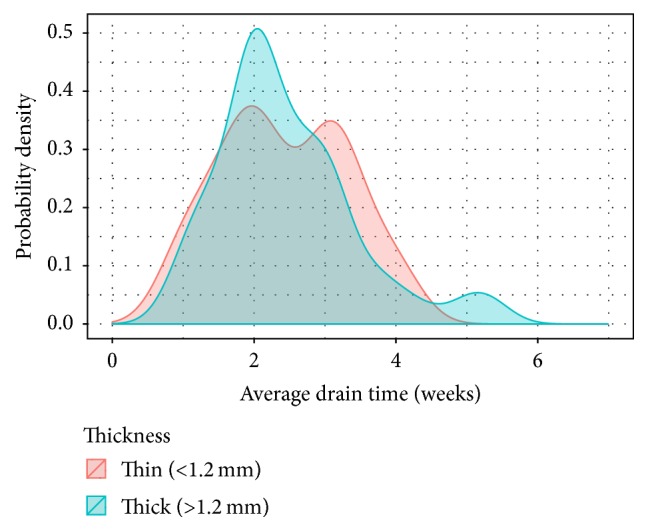


When comparing those with thick versus thin ADMs, those with thick ADMs were more likely to still have drains at the 2-week mark (Figure 1). We observed a positive correlation between thickness of ADMs and average drain time without statistical significance (Figure 1).

**Figure 2:**
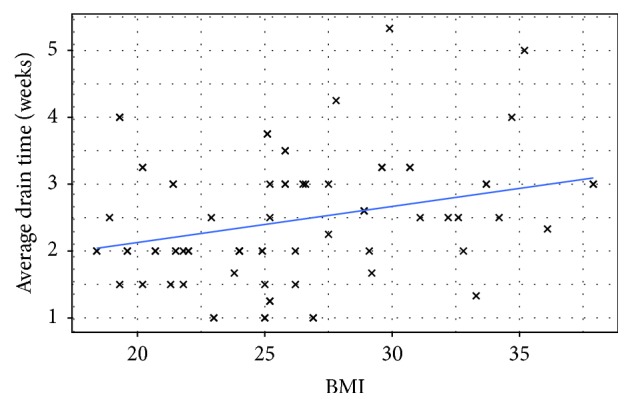


We found statistical significance in patients with higher BMIs to have prolonged drain times (Figure 2). A linear regression model identified BMI as a significant independent predictor for average drain time. One-unit increase in BMI would lead to a 0.0712 ± 0.0225-week increase in average drain time (*p* = 0.002). Also, logistic regression identified radiation (*p* = 0.006) as independent predictor for overall development of complications.

Overall, 42.0% of our patients went on to have autologous reconstruction after expansion, 40.6% had permanent implants placed, 1.4% had TE removal and no reconstruction, and the rest are pending definitive reconstruction.

## 4. Discussion

Despite all of their advantages, studies have linked the use of ADMs in TE based breast reconstruction to higher complication rates compared to total submuscular expander placement. The use of AlloDerm in a series by Chun et al. was associated with a fourfold increase in seroma rate and a fivefold increase in infection rate when compared to the non-ADM group [1]. One-third of patients in another study developed a post-op seroma within 72 hours of drain removal [13]. A meta-analysis performed by Ho et al. showed a higher likelihood of seroma, infection, and reconstructive failure when compared to patients who had a tissue expander with myofascial flap coverage [14]. Other studies, such as the analysis by Vardanian et al., showed no difference between patients with and without ADM in regard to infections or development of seroma/hematoma [15]. Clearly, the literature is divided with regard to the complications associated with TE based breast reconstruction using ADM.

The ability to better identify risk factors for major complications may allow us to choose better candidates to undergo breast reconstruction with an ADM and/or modify risks and technique accordingly [13]. There is a paucity of literature regarding details of complication development, particularly with specific ADM choice and characteristics. However, some characteristics have been ascertained. Fenestrated ADMs theoretically help reduce seroma formation by allowing better effacement of the product against the mastectomy skin, increasing surface area to allow revascularization, and making it easier for fluid to drain [16]. Some studies have suggested that particular ADMs are more prone to complications [16]. Generally, the complication rate between FlexHD and AlloDerm is similar, although Ranganathan et al. found increased infections with FlexHD [17]. Drs. Vu et al. found that using a deep dermal ADM with increased porosity decreased complication rates [18]. The difference between the ready to use (RTU) AlloDerm, which is sterile, and the freeze-dried (FD) AlloDerm, which is aseptic, was also studied and no difference in complication profiles was found between either product [19]. Lastly, appropriately placed closed suction drains and prolonged drainage may help prevent seroma in ADM based prosthetic reconstruction [14].

To our knowledge, this is the first study to consider the complication rates of TE reconstruction with an ADM sling as a function of thickness of the ADM. We noticed a trend toward thicker ADMs being associated with higher complication rates. Patients with thicker ADMs had more seromas (14.6% versus 11.1%), infections (17.1% versus 8.3%), skin necrosis (14.6% versus 11.1%), and need for reintervention (17.1% versus 8.3%). While some of our data failed to reach statistical significance, assessing these complications as a function of increasing thickness was statistically significant. This suggests that ADM thickness is a risk factor for seroma and prolonged JP drain times, but our study was underpowered to reach statistical significance.

While some studies suggest that ADM use increases risk of seroma and complication rate, the exact mechanism remains unknown. Since AlloDerm incorporates into tissue by neovascularization [20, 21], we proposed that the thickness of ADM is directly related to its speed of incorporation and therefore length of drain placement. As with any graft, thicker tissue requires a longer time for incorporation and is at higher risk for failure of integration. Also, drain times were averaged to the nearest week based on available data and presentation to the clinic for removal. Statistical significance may have been achieved if we removed each drain on the exact day when output was less than 30 cc/day. However, for practical reasons, patients could not return to the outpatient setting for drain removal on the exact day when ready for removal.

Reasons for complication development are multifactorial. Poor quality mastectomy skin flaps and smoking are known risks [13]. Common problems are skin flap necrosis, infections, and seromas. A triad of these factors frequently occurs, but their precise relation to each other is yet to be determined [13]. This held true with our patient population, as those with one complication were more likely to have another complication. Increased BMI is a risk factor for seroma, likely due to increased dead space and redundant skin flaps [13]. Seroma may also be attributed to drain specific protocols and the presence of increased dead space where the ADM is placed [15].

Infectious complications are multifactorial; however, the ADM itself can act as a nidus for bacterial colonization and can lead to an infection before the tissue incorporates and revascularizes [3]. Prolonged drain use may also be a risk factor for infection, as the drain can seed an infection [13]. When skin necrosis or breakdown occurs it can lead to infection and eventual exposure [3, 11, 15]. Since seroma rates are higher for ADM based reconstructions, secondary infections are also more frequent. ADM is essentially a foreign body, and the addition of a foreign body with a prosthetic is also an infection risk [15].

Ultimately, it is best to prevent seroma occurrence, but if it develops it needs to be managed appropriately [13]. Many factors, such as patient comorbidities (BMI, diabetes, and smoking) and impaired vascularity of the mastectomy flaps, are outside of the plastic surgeon's control [13]. Some studies have suggested avoiding ADM use in obese patients, where increased dead space and poorly perfused flaps can increase the complication rate [22]. The TE itself can be used to help prevent seroma formation. It is our opinion that the TE should be filled intraoperatively to a point where it approximates the ADM to the mastectomy skin, but without placing excess pressure on the overlying skin, which could lead to ischemia of the mastectomy flaps [3].

One interesting finding in our study is that BMI is an independent risk factor for prolonged drain time. It is well known that patients with increased BMI have a higher complication rate. However, patients with increased BMI and increased breast size are more likely to have infections and mastectomy skin flap necrosis. Likely, this is due to increased dead space and poor apposition of vascularized tissue to the ADM, predisposing it to failure of incorporation.

## 5. Conclusion

Increased BMI was found to be a statistically significant risk factor for maintaining JP drainage for a longer period of time. There is a clear trend toward increased complication rates when thicker ADMs were chosen; however this did not reach statistical significance. Larger, prospective studies comparing those with thick and thin ADMs are warranted in the future for more thorough characterization of associated risks and to quantify an ideal ADM thickness.

## Figures and Tables

**Table 1:** Patients' characteristics comparing thick and thin ADMs.

	Thin ADMs (36)	Thick ADMs (41)	*p* value
Average age (±SD)	50.2 (±12.33)	46.24 (±12.85)	0.2419
Average BMI (±SD)	25.8 (±5.09)	26.1 (±4.90)	0.6321
Number of patients with DM	5 (13.9%)	0 (0%)	**0.0136**
Number of patients requiring radiation	7 (19.4%)	13 (31.7%)	0.2208
Number of smokers	6 (16.7%)	4 (9.8%)	0.3681

**Table 2:** Development of complications comparing thick and thin ADMs.

	Thin ADMs (36)	Thick ADMs (41)	*p* value
Developed seroma	4 (11.1%)	6 (14.6%)	0.6463
Developed hematoma	1 (2.8%)	0 (0%)	0.2827
Developed infection	3 (8.3%)	7 (17.1%)	0.2550
Developed skin necrosis	4 (11.1%)	6 (14.6%)	0.6463
Required intervention	3 (8.3%)	7 (17.1%)	0.2550
Average drain weeks (±SD)	2.43 (±0.9)	2.45 (±1.0)	0.8523

**Table 3:** Characteristics of patients with and without seromas.

	− Seroma (67)	+ Seroma (10)	*p* value
Average age (±SD)	48.0 (±12.81)	48.9 (±12.44)	0.7732
Average BMI (±SD)	26.1 (±4.98)	25.0 (±4.97)	0.5437
Number of patients with DM	5 (7.5%)	0 (0%)	0.3717
Number of patients with radiation	16 (23.9%)	4 (40.0%)	0.2782
Number of smokers	8 (11.9%)	2 (20.0%)	0.4794
Number of thick ADMs	35 (52.2%)	6 (60.0%)	0.6463
Developed hematoma	0 (0%)	1 (10.0%)	**0.0092**
Developed infection	5 (7.5%)	5 (50.0%)	**0.0002**
Developed skin necrosis	6 (9.0%)	4 (40.0%)	**0.0064**
Required intervention	4 (6.0%)	6 (60.0%)	**<0.0001**
Average drain weeks (±SD)	2.28 (±0.8)	3.53 (±1.0)	**0.0004**
